# Marker-free PLRV resistant potato mediated by Cre-*loxP* excision and RNAi

**DOI:** 10.1007/s11248-016-9976-y

**Published:** 2016-08-20

**Authors:** Jeanette Orbegozo, Dennis Solorzano, Wilmer J. Cuellar, Ida Bartolini, Maria Lupe Roman, Marc Ghislain, Jan Kreuze

**Affiliations:** 1 International Potato Center (CIP), P.O. Box 1558, Lima 12, Peru; 2 CIP, P.O. Box 25171, Nairobi, 00603 Kenya; 3 West University Av. C/O Veterinary Service, El Paso, TX 79968 USA; 4 Dirección de Salud (DISA) II, Ministerio de Salud, Lima 04, Peru; 5 International Center for Tropical Agriculture (CIAT), Palmira, Colombia; 6 Servicio Nacional de Sanidad Agraria (SENASA), Lima, Peru

**Keywords:** Potato leaf-roll virus resistance, Marker free, Cre-*loxP*, RNAi

## Abstract

An inverted repeat construct corresponding to a segment of the potato leaf roll virus coat protein gene was created under control of a constitutive promoter and transferred into a transformation vector with a heat inducible Cre-*loxP* system to excise the *nptII* antibiotic resistance marker gene. Fifty-eight transgenic events were evaluated for resistance to PLRV by greenhouse inoculations, which lead to the identification of 7 highly resistant events, of which 4 were extremely resistant. This resistance was also highly effective against accumulation in subsequent tuber generations from inoculated plants, which has not been reported before. Northern blot analysis showed correlation of PLRV specific siRNA accumulation with the level of PLRV resistance. Heat mediated excision of the *nptII* antibiotic resistance gene in PLRV resistant events was highly efficient in one event with full excision in 71 % of treated explants. On the other hand 8 out of 10 analyzed events showed truncated T-DNA insertions lacking one of the two *loxP* sites as determined by PCR and confirmed by sequencing flanking regions in 2 events, suggesting cryptic LB sites in the non-coding region between the *nptII* gene and the flanking *loxP* site. Accordingly, it is proposed to modify the Cre-*loxP* vector by reducing the 1 kb size of the region between *nptII*, *loxP*, and the LB.

**Electronic supplementary material:**

The online version of this article (doi:10.1007/s11248-016-9976-y) contains supplementary material, which is available to authorized users.

## Introduction

Potato leaf roll virus (PLRV, Genus: *Polerovirus*, Family: *Luteoviridae*) is one of the most widespread and important virus disease in potato cultivation (Barker and Dale 2006). In nature, PLRV is obligatorily transmitted in a persistent, circulative and non-propagative manner by aphids (Harrison 1958; Sylvester 1980) and through seed tubers, causing serious economic and yield losses in potato worldwide (Novy et al. 2007). PLRV damages of the potato crop are controlled by the use of certified seed (Radcliffe and Ragsdale 2002) and by insecticide applications (Mowry 2005). However, these techniques are not always accessible, may be also costly, in particular to small-farmers, and produce environmental and human health concerns (Paoletti and Pimentel 2000; Syller 1996; Thomas et al. 1997).

A more economic and environmentally acceptable way to control PLRV is the cultivation of potatoes with enhanced resistance (Barker and Waterhouse 1999). Immunity to PLRV in commercial cultivars has not been reported to date (Taliansky et al. 2003; Thomas et al. 1997). However, different types and sources of resistance have been used by potato breeders. The levels of resistance are classified into two modes of operation: (1) resistance against infection by viruliferous aphids; (2) and resistance to accumulation of the virus (Barker and Harrison 1986). Unfortunately, both resistances until recently were polygenic and their use in breeding programs is time-consuming with an uncertain outcome (Barker and Dale 2006; Davidson 1973; Taliansky et al. 2003). However, a valuable source of resistance in *Solanum tuberosum* Andigena Group was recently found with a major QTL on chromosome V conferring resistance to PLRV and is now introgressed into advanced breeding lines by marker-assisted selection (Mihovilovich et al. 2014; Velásquez et al. 2007).

The direct transfer of PLRV resistance genes into widely grown varieties by genetic transformation presents the advantage of maintaining the variety unchanged except for the introduced trait. The use of transgenes for expression of plant virus sequences, to induce resistance (pathogen derived resistance), is one means by which the problems of complex inheritance of PLRV resistance can be overcome (Barker and Waterhouse 1999), and there are several reports on the successful application of this technology as well as commercial releases of NewLeaf varieties in the US (Barker et al. 1992, 1994; Ehrenfeld et al. 2004; Graham et al. 1997; Kawchuk et al. 1990; Thomas et al. 2000). Pathogen-derived resistance activates a natural cytoplasmic antiviral mechanism called RNA interference (RNAi). In RNAi, double-stranded RNA (dsRNA) is recognized in eukaryotic cells and cleaved by an RNase III- like ribonuclease, called *Dicer*, into small RNA of 21–23 bp. These small interfering RNAs (siRNAs) are loaded into an RNA-induced silencing complex (RISC) that then act as guides to recognize complementary RNA which are cleaved again (Waterhouse et al. 2001). Accordingly, the expression of dsRNA via an inverted repeat of viral sequences, separated by a spacer or intron, has been successful to generate protection against viruses by RNAi (Smith et al. 2000; Waterhouse et al. 1998). High levels of resistance in transgenic plants expressing such hairpin construct have been obtained also against potato viruses such as, Potato virus Y, Potato virus X, Potato virus A and Potato leaf roll virus (Arif et al. 2012; Bai et al. 2009; Chung et al. 2013; Missiou et al. 2004).

Furthermore, pathogen-derived resistance has the advantage of not accumulating protein or long stretches of viral RNA (as dsRNA is almost instantly degraded into siRNA). This situation avoids concerns, such as recombination, transcapsidation and synergism between a viral protein produced from a transgene, and an infected plant RNA virus which makes it attractive from a biosafety perspective (Latham and Wilson 2008; Lemgo et al. 2013).

On the other hand, the use of antibiotic resistance genes for the selection of transgenic plants has remained controversial in spite of proof of safety (Miki and McHugh 2004; Ramessar et al. 2007). The Codex Alimentarius suggested the use of alternative technologies, demonstrated to be safe, that do not rely on antibiotic resistance and other selectable markers in GMOs (Alimentarius 2003). Therefore, several alternatives have been developed in order to obtain transgenic plants free of selectable marker gene (Darbani et al. 2007; Hare and Chua 2002; Puchta 2003; Scutt et al. 2002). Among different systems, the Cre-*loxP* from *Escherichia coli* phage P1 recombination system has proven effective using a heat shock inducible promoter (*hsp*) in *Arabidopsis* (Hoff et al. 2001), maize (Zhang et al. 2003), tobacco (Liu et al. 2005), poplar (Fladung et al. 2005) potato (Cuellar et al. 2006) and rice (Khattri et al. 2011). This system expresses the *cre* recombinase to eliminate the antibiotic resistance and the *cre* gene which are flanked by directly repeated *loxP* sites (Dale and Ow 1991; Gilbertson 2003; Hoess and Abremski 1990).

At the same time, the criteria of the Environmental Protection Agency (EPA), Food Drug Administration (FDA), United State Department of Agriculture (USDA) and other important agencies about the control and biosafety of genetically modified organism (GMO) consider that the integrity of T-DNA, insertion copy numbers, frequency of chromosomal rearrangements, the insertion sites, flanking sequences, and T-DNA preference toward particular regions of genes are knowledge required to facilitate the biological safety evaluation (Cullen et al. 2011; McHughen and Smyth 2008). These information have been described in several plants such as *Arabidopsis thaliana* (Hoff et al. 2001), rice (An et al. 2003), tomato (Thomas and Jones 2007), oat (Svitashev et al. 2000), cotton (Zhang et al. 2008) and potato (Cullen et al. 2011).

In this study we have used our previously developed heat inducible self-excision by Cre-*loxP* system of the *nptII* selectable maker gene and an inverted repeats of the coat protein (CP) gene of PLRV in a hairpin (hpRNA) to obtain a marker-free transgenic plant with high level of resistance to PLRV. Additionally, we analyzed siRNA production, the T-DNA insertion copy number, the site and their flanking sequence of resistant events by using TAIL-PCR, and Genome walker amplification.

## Materials and methods

### Gene construct with excisable *npt*II gene and hairpin PLRV RNAi

**Fig. 1:**
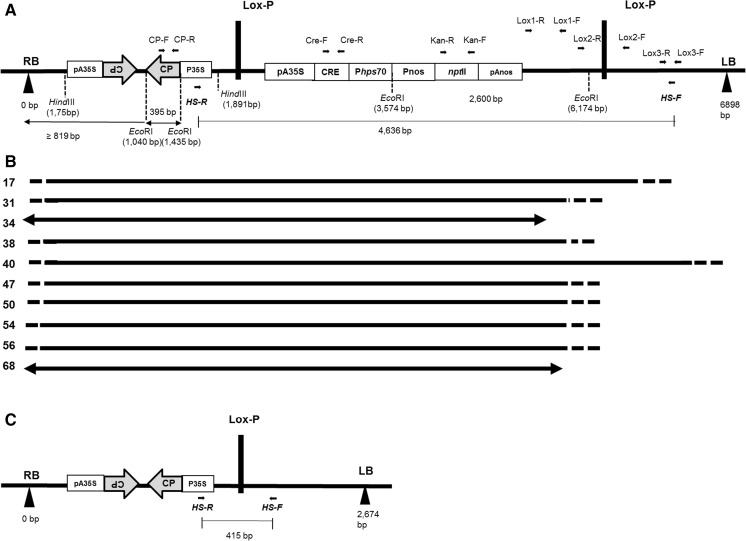
Schematic diagram of the T-DNA region of the pCIP35 carrying the inverted-repeat sequence of CP-PLRV and Cre-*loxP* excisable system under heat-inducible control. **a** T-DNA of 6896 bp from *left to right*: RB right border; pA35S is the CaMV35S poly-adenylation signal sequence; coat protein (CP) gene of PLRV (395 bp) with inverted repeat separated by the IV2 intron; P35S CaMV35S promoter; *loxP* represents the recombination sites; CRE represents *cre* recombinase gene; P*hps*70 is the heat-inducible promoter; Pnos is the nos promoter; *npt*II represents the antibiotic resistance gene used a selectable marker; pAnos is the nos poly-adenylation signal sequence; LB, left border. *Hin*dIII and *Eco*RI are restriction sites with position relative to first nucleotide of the RB, indicated by *dotted line*. Approximate positions of amplification primers are indicated by *short thick arrows*. The *thin arrow bands* indicate the minimum size of the positive band by Southern blotting using conditions described in materials and methods. The *thin line* flanked by HS-F/HS-R primers indicates the expected amplicon including the Cre-*loxP* self-excisable system. **b** The *lines with dashed ends* indicate the approximate inserted fragment of T-DNA as determined by PCR for each event as indicated to the *left*. *Arrowheads* indicate exact position of fragment ends were determined by sequencing. **c** T-DNA structure after successful excision of the nptII gene

A fragment of 395 bp from the coat protein encoding sequence from PLRV (cpPLRV) 3626–4034 position of GenBank accession D13953.1 was used to construct an intron spliced hpRNA. This sequence was cloned in sense and antisense orientation, separated by the IV2 intron (217 bp; Vancanneyt et al. 1990) under the control of the Cauliflower Mosaic virus 35S promoter and CaMV35S poly-adenylation signal sequence using standard molecular techniques (Sambrook and Russell 2001). This gene cassette (referred to as hpPLRV) was inserted into the *Hin*dIII site of the *nptII* heat inducible self-excision vector pCIP33 (Cuellar et al. 2006) to produce the binary vector pCIP35 bearing a T-DNA with the hpPLRV, the *nptII* selectable marker gene, and the Cre-*loxP* excisable selectable marker system whose which complete size was 6896 bp (Fig. 1a). This plasmid was transferred into *Agrobacterium tumefaciens* hypervirulent strain EHA105 (Hood et al. 1993) following the protocol of electroporation (Sambrook and Russell 2001).

### Potato genetic transformation

Pathogen-free plants of the potato variety ‘Desiree’ (accession number CIP800048) were obtained in vitro from the Gene Bank at the International Potato Center (CIP). Plantlets were cultured in liquid propagation medium (4.3 g/L Murashige and Skoog (MS) salts, 0.4 mg/L thiamine, 2 mg/L glycine, 0.5 mg/L nicotinic acid, 0.5 mg/L pyridoxine, 0.1 mg/L gibberellic acid, 2 % sucrose, and pH 5.6) during 4 weeks in a growth room at 14–16 °C, 70 % relative humidity, and 16 h photoperiod (2,000 lux).

In parallel, *A. tumefaciens* carrying the plasmid pCIP35 was cultivated on Luria–Bertani (LB; 1 % bacto-tryptone, 0.5 % yeast extract, 1 % NaCl, pH 7.5, and 2.5 g/L of agar) supplemented with 100 mg/L kanamycin at 28 °C for 48 h. A single bacterial colony was used to inoculate 3 ml of liquid media LB that contained 100 mg/L kanamycin, and 100 mg/L rifampicin at 28 °C for 20 h in a water bath shaker (250 rpm). Leaves with the petiole (1.5–2 cm) were cut from the top third of the in vitro plantlets and co-cultivated with *A. tumefaciens* at a final concentration of 5 × 10^7^ cells/ml (bacterial concentration was estimated from standard curve by absorbance at 620 nm) in 25 ml of co-culture medium (4.6 g/L of MS salts, 2 % sucrose, and 50 μM acetosyringone) at 22 °C for 24 h in dark. The explants were then taken out from liquid medium and briefly dried on sterile filter paper before transferring them to semi-solid regeneration medium (4.6 g/L MS salts, 0.02 mg/L gibberellic acid, 0.02 mg/L naphthalene acetic acid, 2 mg/L zeatin riboside, 2 % sucrose, pH 5.6 and 4 g/L of agar) without antibiotic for one day. Subsequently they were transferred (ten leaves per petri dish) to semi-solid regeneration medium containing 100 mg/L kanamycin and 200 mg/L carbenicillin and transferred onto fresh medium every 2 weeks. After one month regenerants started to appear from *Agrobacterium*-infected leaf explants, and putative transgenic regenerants (only one regenerant per explant) were screened by PCR to detect cpPLRV (CP-F/R), *nptII* (Km-F/R), and *cre* recombinase (Cre-F/R) genes respectively after 6 weeks of growth. The regenerants were propagated in test tubes containing semi-solid regeneration medium at 15–18 °C under artificial light (16 h light/8 h dark). Individual transgenic events were designated as CIP35-[n]. The regeneration efficiency (%) was calculated as the number of regenerated shoots over the number of *Agrobacterium*-infected explants. Transformation efficiency (%) was based on the number of PCR and Southern blot positive plants over the number of *Agrobacterium*-infected explants.

The leaves from the putative transgenic regenerants were confirmed to be transgenic on a highly selective medium. Leaves were laid on kanamycin selective callus-inducing medium (4.6 g/L MS salts, 20 g/L D-mannitol, 0.5 g/L 2-(*N*-morpholino)-ethane sulfonic acid, 0.5 g/L polyvinylpyrrolidone (40,000), 200 mg/L l-glutamine, 40 mg/L adenine, 0.1 mg/L naphthalene acetic acid, 0.1 mg/L 6-benzylaminopurine, 0.5 mg/L nicotinic acid, 0.5 mg/L pyridoxine, 2 mg/L glycine, 2 % sucrose, adjusted to pH 5.8, and 2 g/L gelrite). Then, the sterilized medium was supplemented with 1 mg/L zeatin riboside, 0.1 mg/L naphthalene acetic acid and 200 mg/L kanamycin. Untransformed potato leaf from the varieties ‘Desiree’ were included as controls. After one month, callus appeared only from positive transgenic events.

### Plant nucleic acid isolation

For DNA isolation, we used 4 g of fresh leaf tissue ground in liquid nitrogen. The fine powder was transferred into tubes with 20 ml of CTAB extraction buffer (100 mM Tris–HCl, 25 mM EDTA, 1.4 M NaCl and 2 % CTAB) and 200 μl of 2-mercaptoethanol and incubated at 65 °C in a water bath for 20 min, and cooled down to room temperature (RT) for 10 min. Then, 20 ml of chloroform: isoamyl alcohol (24:1) was added, and mixed gently for 5 min. The extract was centrifuged at 5,000 rpm for 20 min. The upper phase was transferred to a clean 50 ml tube, and this step was repeated one more time. Then 15 ml of cold isopropanol was added and mixed well by inversion, and incubated at −20 °C for 1 h, or −70 °C for 30 min. Precipitated DNA was centrifuged at 5000 rpm for 20 min. The supernatant was discarded and the pellet left drying out for 5 min. The precipitated DNA was transferred into a clean standard 1.5 ml microtube, washed by adding 300 µl of washing solution I (EtOH 75 % and sodium acetate 0.2 M), and left at room temperature for 10 min. Finally, the DNA was transferred into a clean standard 1.5 ml microtube, washed with 300 µl of washing solution II (EtOH 75 % and 10 mM ammonium acetate), and left at room temperature for 1 min. The pellet was then dissolved in 100 μl of Nuclease-free water and treated with 1 μl of 10 mg/ml RNAse for 15 min at 37 °C. The quality of the DNA extractions was confirmed under UV light by standard TBE agarose gel electrophoresis (1 %) and staining with ethidium bromide. Concentration and purity of nucleic acids were estimated by measuring A^260^ and A^280^ values with a UV spectrophotometer (Sambrook and Russell 2001).

### PCR assay

We designed seven primer pairs in order to amplify various regions of the T-DNA (Table 1). PCR was performed using 1 µl of DNA (100 ng/µl) with the following reagents: 1.5 µl PCR 10× standard *Taq* Reaction Buffer (10 mM Tris–HCl, 50 mM KCl, 1.5 mM MgCl_2_, pH 8.3 at 25 °C), 0.5 µl dNTPs (5 mM), 0.75 µl each of the PCR primers (50 µM), 10 µl water and 0.5 µl *Taq* DNA Polymerase (5000 units/ml Bio Labs). PCR amplification conditions were optimized with an initial denaturation at 94 °C for 1 min followed by 35 cycles of 1 min at 94 °C, the annealing temperature varied according to the primer combination used for 50 s and 72 °C for 1.2 min. Finally, an extension time was added at 72 °C for 7 min, and the PCR reaction was then stored at 4 °C. PCR reactions (15 µl) were analyzed by agarose gel electrophoresis as described above.

**Table 1:** Primer sequences their respective annealing temperature (T_a_) and product size (bp)

Primer	Sequences	T_a_ (°C)	Product size (bp)
CP-F	5′-GAAGACGTAGAAGAGGAGGCAA-3′	60	279
CP-R	5′-TGCAATGGGGGTCCAACTCATAA-3′		
Kan-F	5′-CAGCAATATCACGGGTAGCCA-3′	56	638
Kan-R	5′-GGCTATTCGGCTATGACTGGG-3′		
Cre-F	5′-GCGCGGTCTGGCAGTAAAAA-3′	54	751
Cre-R	5′-ACTATCTAGATTACGGATAT -3′		
Lox1-F	5′-GACATTCAACCGATTGAGGG-3′	55	774
Lox1-R	5′-CGACTGCCCAGGCAAGACCG-3′		
Lox2-F	5′-GGATGTGCTGCAAGGCGATTA-3′	54	572
Lox2-R	5′-GCTACTGATTACGGTGCTGC-3′		
Lox3-F	5′-CGAACGTGGCGAGAAAGGAAG-3′	54	110
Lox3-R	5′-AGCTGGCGTAATAGCGAAGA-3′		
HS-F	5′-GAACGTGGCGAGAAAGGAAGG-3′	56	4,636/415
HS-R	5′-TCGATGAAGTGACAGATAGCTGG-3′		

CP-F/R and KM-F/R were used for amplifying the probe for Southern blotting. The HS-F/R produced two amplicon sizes depending on successful excision event

### Southern blotting hybridization

Genomic DNA (15 μg) was digested with *Eco*RI (20 units) in 150 µl reaction volume, at 37 °C overnight. DNA fragments were separated on a 0.8 % agarose gel electrophoresis overnight, and transferred to nylon membranes (Hybond-N+, Amersham) by capillary transfer according to Sambrook and Russell (2001). DNA fragments were bound to the membrane by UV cross- linking (Southern Stratalinker 2400). cpPLRV gene (279 bp) was used as probe labeled with [α32] dCTP using the Gene Images Random Prime Labeling kit according to the manufacturer’s recommendation. The probes were hybridized with the membranes at 65 °C for 18 h. The hybridized filter was washed with 1x SSC, 0.1 % SDS (w/v) and 0.5 × SSC, 0.1 % SDS (w/v) at 65 °C for 15 min with gentle agitation. The blot was wrapped in a polyethylene sheet and exposed to radiographic film (Kodak). To detect excision events, we used as probe the *nptII* gene (638 bp) labeled with PCR DIG Synthesis (Roche kit) according to the manufacturer’s conditions. The probe was hybridized at 65 °C for 30 min with 15 ml of DIG Easy Hyb solution (Roche kit) at 25 ng/mL. The membrane was washed using stringent conditions and exposed to X-ray film (Kodak) at room temperature for 16 h.

### PLRV resistance assays

Transgenic events and a non-transgenic control were transferred to the biosafety greenhouse to acclimatize and produce tubers. Plants were grown from four tubers from each transgenic event. Thirty days after planting, these events were inoculated by grafting with PLRV infected Flor Blanca scions (Peruvian cultivar highly susceptible to PLRV), or for the case of the non-inoculated control with healthy Flor Blanca. The grafts were secured with Parafilm M (Sigma), and the whole plants were covered with a thin transparent plastic bag to avoid dehydration of the scions. The bag was removed 5 days after grafting. The controls were a non-transgenic Desiree inoculated similarly (positive control), and a mock-inoculated transgenic health plant (negative control). All plants were left to produce tubers and one tuber from each plant was then planted after breaking of dormancy to evaluate resistance to secondary infection. Symptoms were monitored throughout the growing period and scored on a scale from 0 to 2 (0 = no symptoms, 1 = mild symptoms, and 2 = severe symptoms). Based on the results, 10 transgenic events were selected for a repeated experiment, which was carried out as described above, except that 15 plants per event were evaluated.

### Quantification of PLRV by immunological assay

Detection of PLRV in inoculated plants was performed by double antibody sandwich enzyme-linked immunoabsorbent assay (DAS-ELISA). Virus-specific polyclonal antibodies (dilution, 1:1000) to the coat protein of PLRV, and the specific alkaline phosphatase conjugated polyclonal antibodies (dilution, 1:1000) were provided by the Laboratory of Virology at CIP. Absorbance at 405 nm was used to quantify PLRV, 15–30 min after adding the substrate (*p*-nitrophenyl phosphate) according to manufacturers’ instruction of the alkaline phosphatase substrate kit (Bio-Rad), using a microplate reader Bio-Rad 550 (Bio-Rad). A plant was considered infected if the absorbance was higher than the average of the non-infected control plus two times the standard deviation. Results were subjected to statistical analysis using the non-parametric Kruskal–Wallis test (Kruskal and Wallis 1952). The presence of virus was determined at 30, 60 and 90 days after grafting (primary infection) and at 30, 60 and 90 days after planting of tubers from infected plants in secondary infection. All experiments were performed using complete randomized block design, and samples were taken by combining three leaflets (top, middle, and bottom of the plant) per plant, and two individual measurements were made per sample.

### Northern blotting hybridization

Leaf tissue was ground into a fine powder in liquid nitrogen and RNA was isolated with TRIzol LS Reagent (Invitrogen, Ltd.) according to the manufacturer’s instructions. The low-molecular-weight (LMW) RNA fraction containing siRNA was separated by the LiCl_4_ precipitation method as described previously (Kreuze et al. 2005). To analyze siRNA, 30 μg of LMW RNA was mixed with an equal volume of Tris–borate-EDTA– urea sample buffer (Bio-Rad), heated at 100 °C for 5 min and separated on a 15 % polyacrylamide Tris–borate—EDTA—urea gel and blotted to a nylon membrane (Hybond-NX, Amersham). The radiolabeled RNA probe was hybridized overnight, then the membrane was washed three-time with 5 × SSC, 0.1 % SDS (w/v) at 37 °C for 15 min with gentle agitation. The membrane was UV cross-linked as described above, the blots were wrapped in a polyethylene sheet and exposed to a radiographic film (Kodak). Sense and antisense [α32P] UTP labeled RNA probes complementary to CP transgene were synthesized with T7 RNA polymerase (Promega, Madison, WI, USA) using the plasmid pGEM^®^-T Easy linearized with *Nde*I. For the hybridization with siRNA, the probe was denatured by alkaline hydrolysis in carbonate buffer (120 mM Na_2_CO_3_, 80 mM NaHCO_3_, pH 10.2) to obtain fragments of an average length of 50 bp and then hybridized, washed and developed as described by Kreuze et al. (2005). A control sample of siRNA for the hpPLRV was prepared as follows. The *Agrobacterium tumefaciens* bearing the hpPLRV construct was grown overnight at 28 °C in 100 ml of liquid medium LB containing 100 mg/L kanamycin and 50 mg/L rifampicin. The bacterial cells were harvested by centrifugation at room temperature at 4000 rpm for 20 min. Then, these were suspended in a buffer solution (10 mM MgCl_2_ and 150 µM acetosyringone). The suspension of cells was 0.5 of optical density (OD) at 600 nm. After, this mix was left at room temperature for 3 h without shaking. The agroinoculation was pressure-infiltrated on the underside of the leaves of the wild type *Nicotiana benthamiana* cv. using a 2-ml syringe without needle. Three days later, we collected the leaf to isolated siRNA, this sample was used as positive control in the northern blot.

### Excision of the *npt*II gene by heat treatments

Leaves with petioles and stem internodes of transgenic plants were excised and placed on semi-solid regeneration medium without antibiotic selection. Then, these petri dishes were transferred into an incubation oven at 42 °C for 3 h in the dark as described by Cuellar et al. (2006). The explants were then cultivated at 18–23 °C with a photoperiod of 16 h light/8 h dark on same medium. As control, leaf and internodes of the same events without heat shock treatment were included. The regenerated shoots obtained were transplanted in tubes containing semi-solid regeneration medium without kanamycin. In order to select the transgenic events which lost the *nptII* gene by excision, we evaluated them using the kanamycin selective callus-inducing medium as described above.

### Identification of T-DNA genomic flanking sequences

We used two methods to characterize the sequences surrounding the extremity of the T-DNA inserts: the thermal asymmetric interlaced PCR (TAIL-PCR); and the genome walker technique of Clontech. The modified thermal asymmetric interlaced (mTAIL)-PCR protocol as described by Sessions et al. (2002) was used to amplify regions flanking of the T-DNA right border (RB) by PCR. We used the following nested specific primers and their respective Tm: RB-1, (5′-CAA ATC ACC AGT CTC TCT CTA CAA ATC TAT CTC TC-3′) with Tm of 65 °C; RB2 (5′-CAG AAT AAT GTG TGA GTA GTT CCC AGA TAA GG-3′) and 64 °C and RB-3 (5′-GTT CTT ATA GGG TTT CGC TCA TGT GTT GAG-3′) and 66 °C. Each one of the two-rounds of mTAIL PCR cycling was performed in MJ Research PTC-200 Thermo Cycler (BC-MJPC200) using Go-Taq polymerase (5 U/µl) (Promega), and 5 ng of genomic DNA.

In order to amplify regions flanking of the T-DNA left border, we developed three libraries of DNA (100 ng/μl) using *Dra*I (10 units/μl), *Eco*RV (10 units/μl), and *Pvu*II (10 units/μl) enzymes. The ends of the genomic digested fragments were linked with two adaptors (Adaptor Primer 1: 5′-GTA ATA CGA CTC ACT ATA GGG C-3′, and Nested Adaptor Primer 2: 5′-ACT ATA GGG CAC GCG TGG T-3′). PCR conditions followed the protocol of Genome Walker^TM^ Universal kit (Clontech), and used PfuUltra™ II Fusion HS DNA Polymerase (Stratagene). The gene specific primers were derived from sequences close to the end of the known left border (LB) sequence: LB-1 (5′-GTG TCA TCT ATG TTA CTA GAT CGG GCC TCC-3′) with Tm of 67 °C; LB-2 (5′-GCT ACT GAT TAC GGT GCT GCT ATC GAT GGT-3′) and 68 °C; LB-3 (5′-CGG TGA CGG TGA TAA TTC AC-3′) and 57 °C; and LB-4 (5′-CCC TCA ATC GG TTG AAT GTC-3′) and 59 °C. PCR amplifications were performed using MultiGene Thermal Cycler TC9600-G (LabNet).

PCR products were purified from gel using the Wizard gel extraction kit (Promega) according to the manufacturer’s instructions, and sent for sequencing to Macrogen (Korea) with the corresponding PCR primers. Similarity searches for the identified sequences were done by using BLAST at the National Center for Biotechnology Information (http://www.ncbi.nlm.nih.gov).

## Results

### Hairpin PLRV gene construct with excisable *nptII* gene

We chose a fragment of the coat protein gene of PLRV which showed 97–100 % identity with sequences available in the genebank, including isolates from France (Accession number AF453390.1), Spain (Accession number 453393.1); Zimbabwe (Accession number AF453388.1), Peru (AF453392.1) and China (Accession number AY079210.1). It confirmed the high level of conservation of this gene among isolates from geographically distant locations. Moreover, this sequence had 80–90 % similarity with other members from *Luteoviridae* family such as Barley Yellow Dwarf Virus (Accession number X17259.1), Cereal Yellow Dwarf Virus (Accession number AF235168.2), Soybean Dwarf Virus (Accession number DQ145545.1). Thus, a hairpin gene construct using this cpPLRV sequence was designed, and inserted into the corresponding sites of pCIP33 vector to produce the binary vector pCIP35 which contained the kanamycin selection marker gene and the Cre-*loxP* excisable selectable marker system. The correct position and size of several genes from T-DNA were evaluated using four restriction enzymes (*Bam*HI, *Eco*RI, *Hin*dIII, and *Nhe*I), each generating characteristic fragments of the expected sizes (data not shown).

### Genetic transformation and analysis of regenerants

160 potato plants explants were transformed by *A. tumefaciens* with the pCIP35 vector binary, from which 102 explants yielded calli leading to the isolation of 85 regenerated shoots. However, only 55 were positive by kanamycin selective callus-inducing medium with highly selective conditions and by PCR using specific primers to the cpPLRV, *nptII*, and *cre* genes (Fig. 1a). The regeneration efficiency was thus 53 % and transformation efficiency was 34 %. Regenerated potato plants showed no visible phenotypic alteration due to the insertion of PLRV coat protein gene.

### Virus resistance in transgenic plants following PLRV infection

**Fig. 2:**
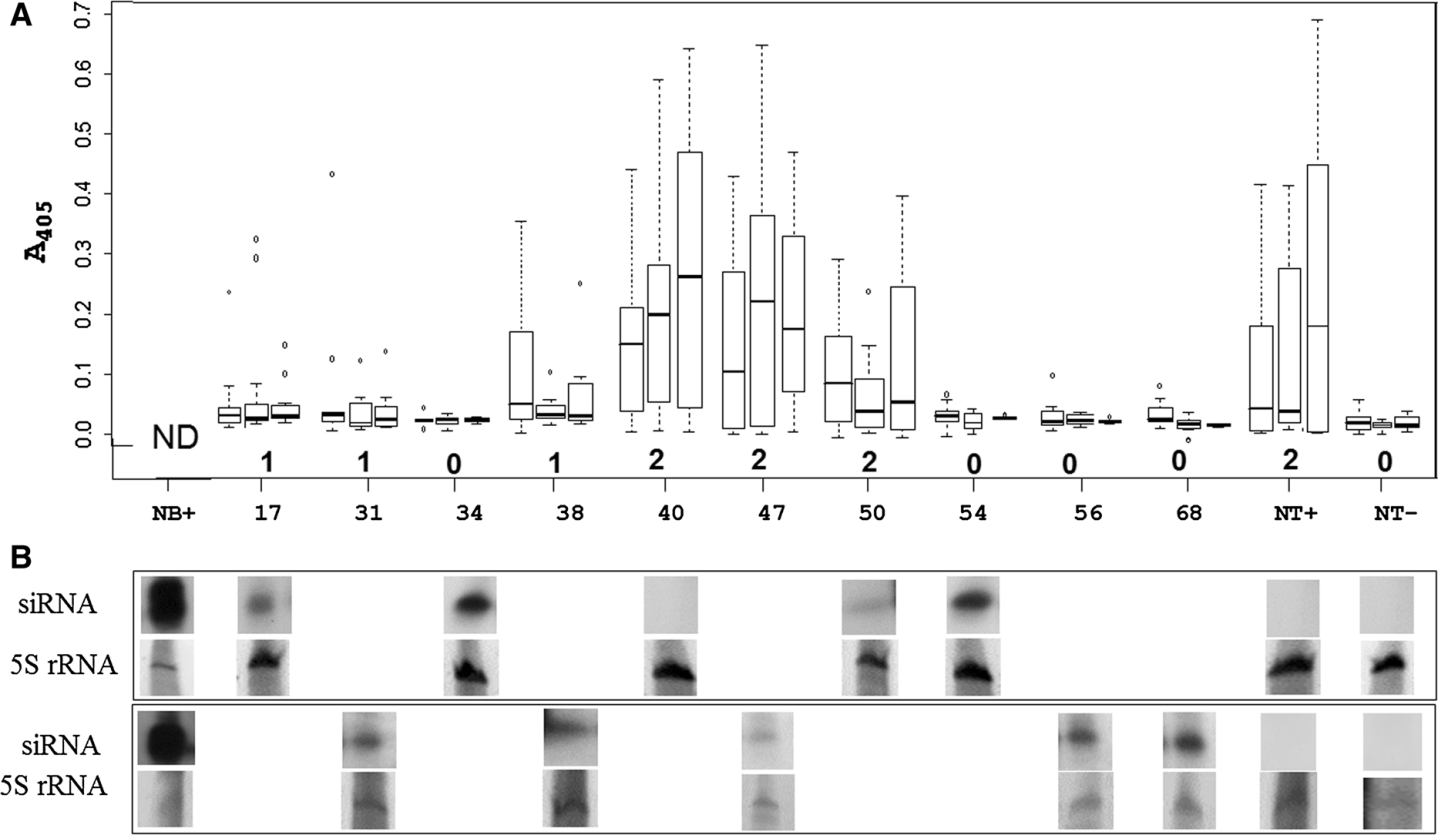
DAS-ELISA and Northern blot. **a** Box-plot graphics showing dispersions and medians of the DAS-ELISA absorbance values (A_405_) of ten transgenic events (17, 31, 34, 38, 40, 47, 50, 54, 56 and 68) measured at three time points (30, 60 and 90 days) after planting of tubers from PLRV inoculated mother plants. Ten transgenic events are shown at x-axis and their absorbance values on y-axis, *open dots* indicate extreme values by Kruskal test.Average symptom severities are shown by *numbers below* each event and controls. Values were obtained from 15 individual plants (replications) of each event. ND not done. NB+: *Nicotiana benthamiana* siRNA control NT−: non-transgenic healthy Desiree; NT+: non-transgenic PLRV infected Desiree. **b** Northern blot analysis of siRNA in ten transgenic non- inoculated *lines* using a probe specific to the hpPLRV and three controls. The composite figure shows siRNA hybridization signal above, and 5S RNA staining by ethidium bromide and visualized under UV light below, for each event and controls. Pictures are grouped according to two different membranes (*above* and *below*) that they were run on and are in the same order as samples in (**a**)

**Fig. 3:**
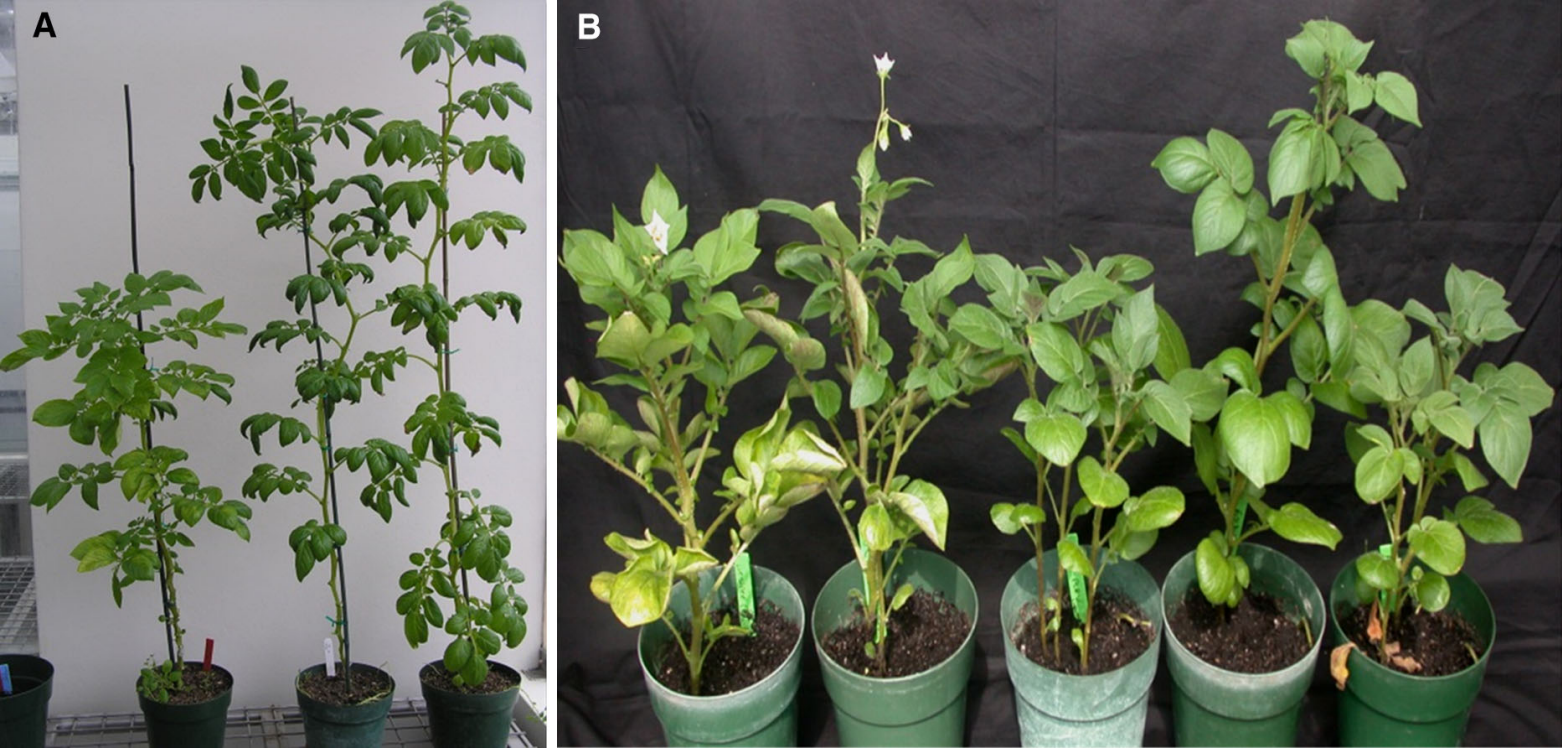
**a** Symptoms of primary infection of PLRV at 60 days post inoculation, from *left to right*: non-transgenic Desiree, resistant event 68, and non-inoculated Desiree. **b** Symptoms of secondary infection of PLRV 30 days post planting of tubers of graft inoculated mother plants, from *left to right*: non-transgenic Desiree, event 40 (non-resistant), event 34 (resistant), event 54 (resistant), and non-inoculated Desiree

Out of the 55 transgenic events transferred to the greenhouse, 53 produced sufficient tubers to perform the PLRV inoculation experiment. Four replicates of each of the 53 events were evaluated for resistance to PLRV by performing DAS-ELISA tests at 30, 60 and 90 days post inoculation. We identified four events that never provided a positive result indicating the presence of PLRV in any of the replicates or time points. However, we found that the number of plants testing negative for PLRV in all the 4 replicates of transformed events increased over time: at 30, 60 and 90 days: the number of events testing negative by DAS-ELISA were 11, 18 and 41 respectively. One tuber per inoculated plant was planted to evaluate resistance to secondary infection by DAS-ELISA at 30, 60 and 90 days after planting. DAS-ELISA results from secondary infection resulted in a larger number of positive samples, and the tendency to recover from infection as found in primary infection was not apparent, indicating that resistance was not sufficient to suppress the more severe secondary infection in the vast majority of these events. Ten events however maintained significantly reduced or negative ELISA values as compared to the control and also remained without symptoms, and were selected for re-evaluation in a second experiment using 15 plants each. All ten events (17, 31, 34, 38, 40, 47, 50, 54, 56 and 68) showed significantly reduced titers during primary infection mostly less than 50 % of the non-transformed control. Again a clear reduction of viral titers was observed as the plants matured indicating recovery, also in the non-transgenic control (Figure S1). Symptoms of stunting and yellowing could be observed in non-transformed cv. Desiree as well as some plants of events 40, 47 and 50 which simultaneously showed the highest DAS-ELISA values among the transgenic events. During secondary infection three different symptoms and response by DAS-ELISA in transgenic plants were observed, when compared to the control plants. The first phenotype was that of plants susceptible to PLRV infection which developed strong virus symptoms similar to inoculated control plants and no indication of recovery (symptom severity value 2) but rather an increase in viral titers (events: 40, 47 and 50), plants in another category showed reduced titers and symptoms (with average symptom value 1; events: 17, 31, 38), and a third category consisted of plants that never showed titers significantly above the untransformed and uninfected control and no symptoms (symptom value 0, events: 34, 54, 56 and 68; Fig. 2a and 3). The four candidate highly resistant transgenic events showed similar results in DAS-ELISA during tertiary infection (data not shown). Average symptom severity correlated well with virus titers, and ELISA negative plants never showed any symptoms at all. The non-parametric Kruskal–Wallis test detected significant differences in virus titers between four resistant events (34, 54, 56 and 68) and non-transgenic infected control (NT+) (Fig. 2a).

### Analysis of T-DNA copy number

**Fig. 4:**
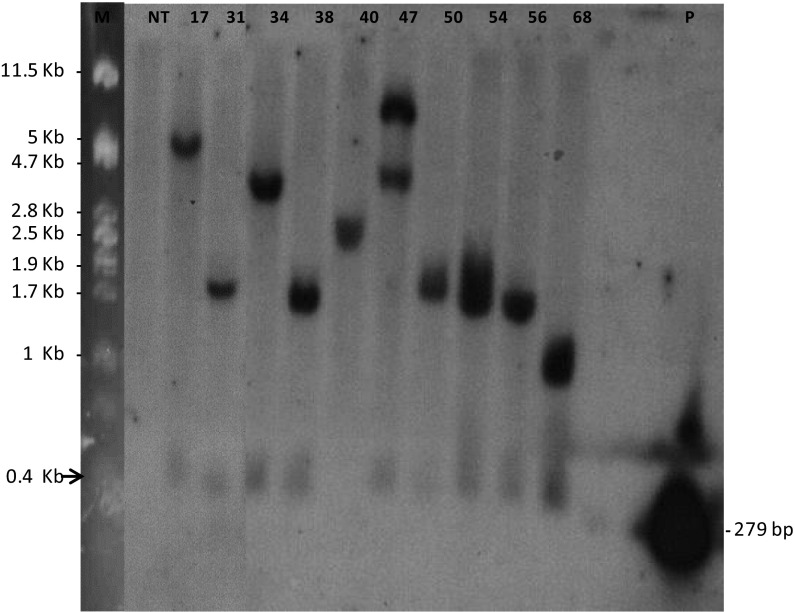
Southern-blot analysis of genomic DNA of the ten best transgenic events. DNA was digested with *Eco*RI and hybridized with a probe corresponding to the coat protein CP of PLRV, resulting in two bands, one corresponding to the 400 bp fragment from sense arm of the CP hairpin (indicated by the *arrow*), and another of variable size corresponding to the antisense arm of the hairpin up to the next restriction site at the insertion site in the potato DNA. M: λ DNA (Gibco-BRL) digested with *Pst*I, NT: Non-transgenic var. Desiree, P: Probe coat protein (CP) of PLRV (279 bp). One lane containing a duplicated sample 34 was removed electronically from the presented figure between current *lanes* with samples 31 and 34

53 transgenic events were subjected to Southern blotting. The restriction enzyme *Eco*RI cuts four times within the T-DNA of pCIP35 (Fig. 1a). The cpPLRV probe hybridized to at least two fragments: (1) a small fragment of 395 bp; and (2) a second fragment of variable size and number which gives an estimate of the T-DNA copy number Fig. 1a. Accordingly, we determined the number of T-DNA inserts to be from 1 to 8 in the 53 transgenic events (data not shown). The band patterns were distinct for each transgenic event which indicated that these were the result of independent transformation occurrences. Eight of the ten selected events for repeated evaluation had one copy of T-DNA whereas the event 47 had two copies and event 54 had one or two copies (Fig. 4). Transgenic event 40 did not have the small fragment suggesting an incomplete or rearranged T-DNA structure around the hairpin gene.

### Detection of siRNAs in the transgenic events

The main feature of the RNAi mechanism in plants is the production of siRNA whose detection and abundance in transgenic plants may predict virus resistance (Missiou et al. 2004). The 10 transgenic events selected for re-evaluation were analyzed approximately 2 months after their transfer to the greenhouse. Northern blot analysis revealed strong hybridization signals corresponding to the size of siRNA in healthy plants of the events: 17, 31, 34, 38, 54, 56, and 68 whereas the events 47 and 50 had low levels only slightly higher than the non-transgenic un-infected plants, and event 40 showed no hybridization signal at all (Fig. 2b).

### Characterization of the *nptII* excision via the Cre-*loxP* system

In order to determine if the ten candidate resistant events had the intact Cre-*loxP* and *nptII* structures, PCR using the primers Lox1-F/R, Lox2-F/R, and Lox3-F/R were used to amplify the *loxP* region near the left border (Fig. 1a). Only the events 17 and 40 were positive using Lox2-F/R and Lox 3-F/R primer pairs respectively, whereas the events 34 and 68 were PCR negative for all three primer pairs and the remaining events were PCR positive only for the Lox1-F/R primer pairs. Hence, only the transgenic events 17 and 40 had both *loxP* sites intact, whereas the other eight transgenic events seemed to be lacking the LB proximal *loxP* site (Fig. 1b). In order to verify the absence of *loxP* site determined by the PCR analyses, we characterized the flanking sequences of two of the most resistant events through genome walking. The sequence towards the LB in the event 34 corresponded to the T-DNA until nucleotide 5448 after which the sequence showed high similarity to potato DNA (*Solanum tuberosum* mechanosensitive ion channel protein 1, mitochondrial-like [LOC 102600077], transcript variant X2, mRNA [Accession number XM_006358602.1] with 90 % nucleotide identity). In the event 68, sequence towards the LB corresponded to the T-DNA until nucleotide 5693 after which it showed high similarity to potato DNA (*Myb* gene sequence from *Solanum tuberosum* [Accession number XP_006355158.1] with 96 % translated amino acid identity). Noteworthy, in both events the insertion has apparently occurred within a potato gene.

### Heat shock treatment of the event resistant to PLRV with intact Cre-*loxPnpt*II structure

Since the transgenic event 40 had apparently a rearrangement around the hairpin gene, produced no detectable siRNA whatsoever and was not resistant, we applied heat shock treatment only to the event 17 because it had an intact Cre-*loxP nptII* structure and was resistant to the PLRV virus. We assumed that the *loxP* site near the right border was complete because these events were positive in Southern blotting for the cpPLRV and PCR positive for the *cre* and *nptII* gene (Fig. 1a).

**Fig. 5:**
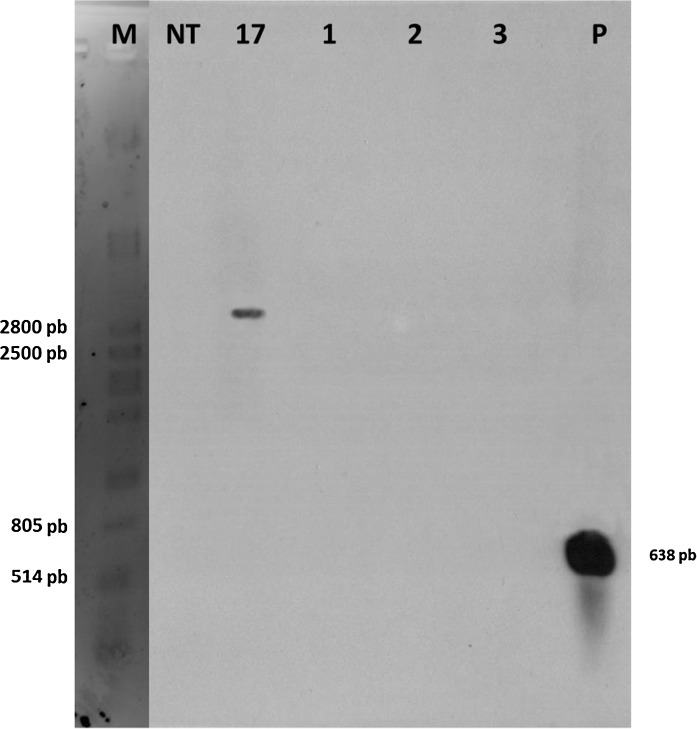
Southern blot analysis of marker excision. M: λ DNA (Gibco-BRL) digested with *Pst*I. NT: non-transformed var. Desiree, 17: transgenic event 17 without heat-shock treatment, *lanes* 1–3: event 17 after heat-shock treatment at 40 °C for 3 h, *P*: probe *npt*II gene

We produced a total of 1501 explants (463 petioles with leaves and 1038 stem internodes) from event 17 and exposed them to heat-shock treatment. After 2 months, we obtained 300 regenerated plants of which 140 did not form callus on kanamycin selective callus-inducing medium. Of these, we evaluated 58 plants in more detail by PCR. Out of these 41 regenerants (41/58 = 71 %) displayed a full excision as determined by PCR using HS-F/HS-R primers (Fig. 1c and S2); 12 regenerants (12/58 = 21 %) appeared to be chimeras, positive with both Cre-F/Cre-R and HS-F/HS-R primers (Figure S3); and 5 regenerants (5/58 = 8 %) did not appear to have had any excision at all being positive with only the Cre-F/Cre-R primers (Figure S4). Southern blot performed on three randomly chosen plants with complete excision confirmed that these did not have the kanamycin resistance *nptII* gene (Fig. 5).

## Discussion

The present work aimed at obtaining PLRV resistant potato plants without an antibiotic resistance gene using RNAi (Waterhouse et al. 1998) and a self-excisable Cre-*loxP nptII* system respectively (Cuellar et al. 2006). RNAi has been used successfully to confer resistance to several plant viruses (Mansoor et al. 2006; Smith et al. 2000; Tenllado et al. 2004) including as PLRV (Arif et al. 2012; Chung et al. 2013). One important criteria is to choose the hairpin sequence with at least 90 % nucleotide identity to the targeted virus (de Haan et al. 1992). Hence, we chose a region of coat protein in the PLRV genome with the least sequence variation in PLRV sequences deposited in GenBank.

50 out of 54 (93 %) of transgenic events showed a recovery phenotype during primary infection: initially significant virus titers reduced during plant maturity resulting in mostly ELISA-negative plants at 90 dpi. The recovery phenotype, typified by initially symptomatic plants recovering from virus infection, is not uncommon among transgenic plants, and it has also previously been observed in PLRV transgenic plants (Ehrenfeld et al. 2004). Recovery was not observed in secondary infection, which is concurrent with the notion that infection is always more severe in plants obtained from infected tubers. In all but ten events virus titers resurged during secondary infection and most plants did not recover, indicating that resistance was not sufficient to suppress the more severe secondary infection in these events. The same was observed in the second experiment using the ten best events: most of the transgenic lines remained completely symptom-less and with low virus titers during primary infection in contrast to the control, indicating they possessed some level of resistance. Only line 40 displayed some symptoms, and correspondingly had similarly high titers as the control at 60 and 90 dpi (Figure S1). During secondary infection however, only four events (34, 54, 56 and 68) never showed symptoms, and remained entirely ELISA negative during secondary infection. Because events 40 and 47 became highly symptomatic and had viral titers similar to the non-transgenic control, it seems that the level of resistance is not necessarily stable in all events, as events 40 and 47 which were selected based on their resistance in the first experiment.

At first it may seem surprising that such a limited number of plants displayed an immune phenotype as several reports indicate a high number of transgenic events providing immunity using similar technology (Kalantidis et al. 2002; Missiou et al. 2004; Smith et al. 2000). However upon closer inspection of the literature it appears that the highest efficiencies are obtained for resistance to potyviruses, and that in the attempts to engineer resistance to luteoviruses (Tougou et al. 2006; Wang et al. 2000), full immunity has never been demonstrated, while number of resistant plants among the transformants was much lower (Chung et al. 2013; Wang et al. 2000). Moreover, as the other crops, previously engineered for luteovirus resistance were seed propagated, clonal generations are irrelevant and were not evaluated. It may be possible that much higher resistance levels are required to control luteoviruses over clonal generations; indeed, even in the best NewLeaf varieties virus infection became detectable in progeny plants from tubers produced from field inoculated plants in which virus was undetectable (Thomas et al. 2000). This was not tested in any of the other reports on transgenic PLRV resistance.

siRNAs were detected by northern blot in all ten selected events except for the most susceptible one (40), and relative amounts were positively correlated with the level of resistance, i.e. the most resistant events had the strongest siRNA signal (Fig. 2). A similar observation in terms of quantities of siRNA was reported by Missiou et al. (2004). Interestingly, siRNAs corresponding to the PLRV CP region could not be detected in non-transgenic plants infected with PLRV. This may be due to PLRV being phloem limited; phloem cells only representing ~1 % of all plant cells and thus if siRNA is produced exclusively in infected cells, their overall quantity may fall below the detection limit, even if they were relatively abundant within those cells. Indeed, we have found PLRV derived siRNAs can be readily detected from infected potato plants by high throughput sequencing, although their relative amounts are extremely low in comparison to other viruses that are not phloem limited such as PVY or PVX (data not shown). The lack of siRNA detected in event 40 is consistent with our inability to detect one of the inverted repeats of the CP hairpin structure.

Upon analyzing, by PCR, the integrity of the T-DNA sequences present in the ten selected events, we were surprised to find that nearly all of them were truncated in their LB region, and that all but two of them had even lost the left *loxP* site. Indeed, original attempts to excise the *Cre*-*nptII* cassette in the most resistant events (34, 54, 56 and 68) failed to provide any kanamycin susceptible regenerants (data not shown). The truncation of the T-DNA fragment before the left *loxP* site was further confirmed by genome walking and sequencing in two of the resistant events, which were able to identify the exact position at which each of them were truncated, about 1200 bp downstream of the LB sequences. Similar large deletions of 900 and 1500 bp have been reported in other studies (Gambino et al. 2009; Tinland 1996) and may be due to the micro-similarities between the T-DNA sequence and the plant genome toward the LB (Gambino et al. 2009), rearrangements during the T-DNA integration (Kohli et al. 2003; Nacry et al. 1998). Indeed, it is known that integration of the LB is less exact during T-DNA insertion into the genome as compared to RB sequence (Meng et al. 2007; Zhang et al. 2008). Whatever the reason, such a high frequency of truncated insertions is undesirable because it renders most transformed events unsuitable for marker gene excision. Improvement of the pCIP35 vector by removing unnecessary sequences so that the *loxP* site tightly follows the *nptII* gene should be able to strongly reduce such truncations effectively.

Transgenic plants which use Cre-*loxP* system can produce different excision results such as complete (Gilbertson 2003), chimerics (Tuteja et al. 2012; Zuo et al. 2001) or no excision at all (Li et al. 2007), and we found similar results in our study. Using this system we obtained 71 % excision efficiency, which is considerably higher to the 4–8 % of excision previously obtained by Cuellar et al. (2006) who used the same backbone construct. Thus we believe that Cre-*loxP* system is an effective mechanism in contrast to selection without marker gene, which is consider very laborious (Bukovinszki et al. 2007) and not efficient (Kumar and Thompson 2009).

In conclusion, we obtained seven events with increased resistant to PLRV, of which four were extremely resistant. This demonstrates extremely high levels of resistance can also be obtained against luteoviruses such as PLRV, which is maintained also over tuber generations, something that had not been demonstrated before. Whereas analysis of the integration sites revealed that most T-DNA insertions had been truncated towards their LB, thereby losing one of the *loxP* sites, highly efficient heat shock mediated excision from resistant event 17, which contained both flanking *loxP* sites, was demonstrated. Thus, if the issue of undesirable truncation of the transgene cassette, by eliminating unnecessary sequences in the *nptII* gene and the left *loxP* site, can be resolved, the pCIP35 vector would provide an excellent vector for selectable marker free introduction of PLRV resistance into susceptible potato varieties. The level of resistance to PLRV in this study was correlated to production of siRNAs in the transgenic plants, thus our data suggest preliminary screening of events based on siRNA production could be applied to rapidly select candidate resistant events for virus inoculation in field or greenhouse. On the other hand, the PLRV resistant Desiree event 17, lacking *npt*II generated in this study is now a candidate to be evaluated for resistance to PLRV under field conditions and could be used to add additional traits of interest by transgenesis.

## Electronic supplementary material

Below is the link to the electronic supplementary material.

Supplementary material 1 (DOCX 809 kb)

Supplementary material 2 (PPTX 72 kb)
