# Supplementary material for: Marker-free PLRV resistant potato mediated by Cre-loxP excision and RNAi
Source: Transgenic Res. 2016 Aug 20;25(6):813–28. doi: 10.1007/s11248-016-9976-y (PMC5104775; doi:10.1007/s11248-016-9976-y)
Supplement: Supplementary file 1 — Supplementary material 1 (DOCX 809 kb) [file 11248_2016_9976_MOESM1_ESM.docx]

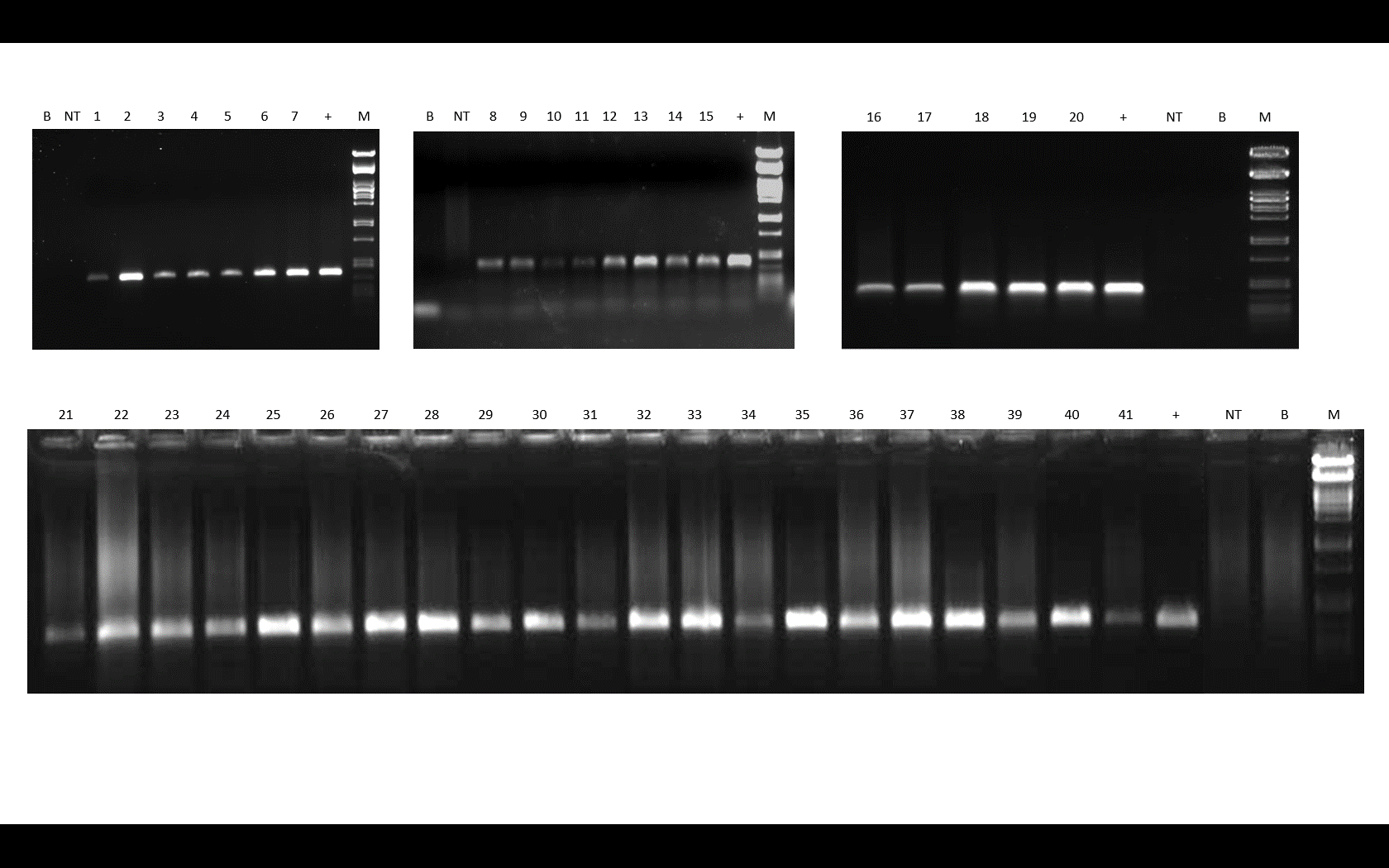


**Figure S2**. PCR analysis of the full excision events by the use of HS-F/HS-R primers. B: blank, NT: non-transgenic Desiree, +: pCIP35, M: λ DNA (Gibco-BRL) digested with *Pst*I, and rests of lanes (1-41) are transgenic lines showing the required amplification (415 bp).


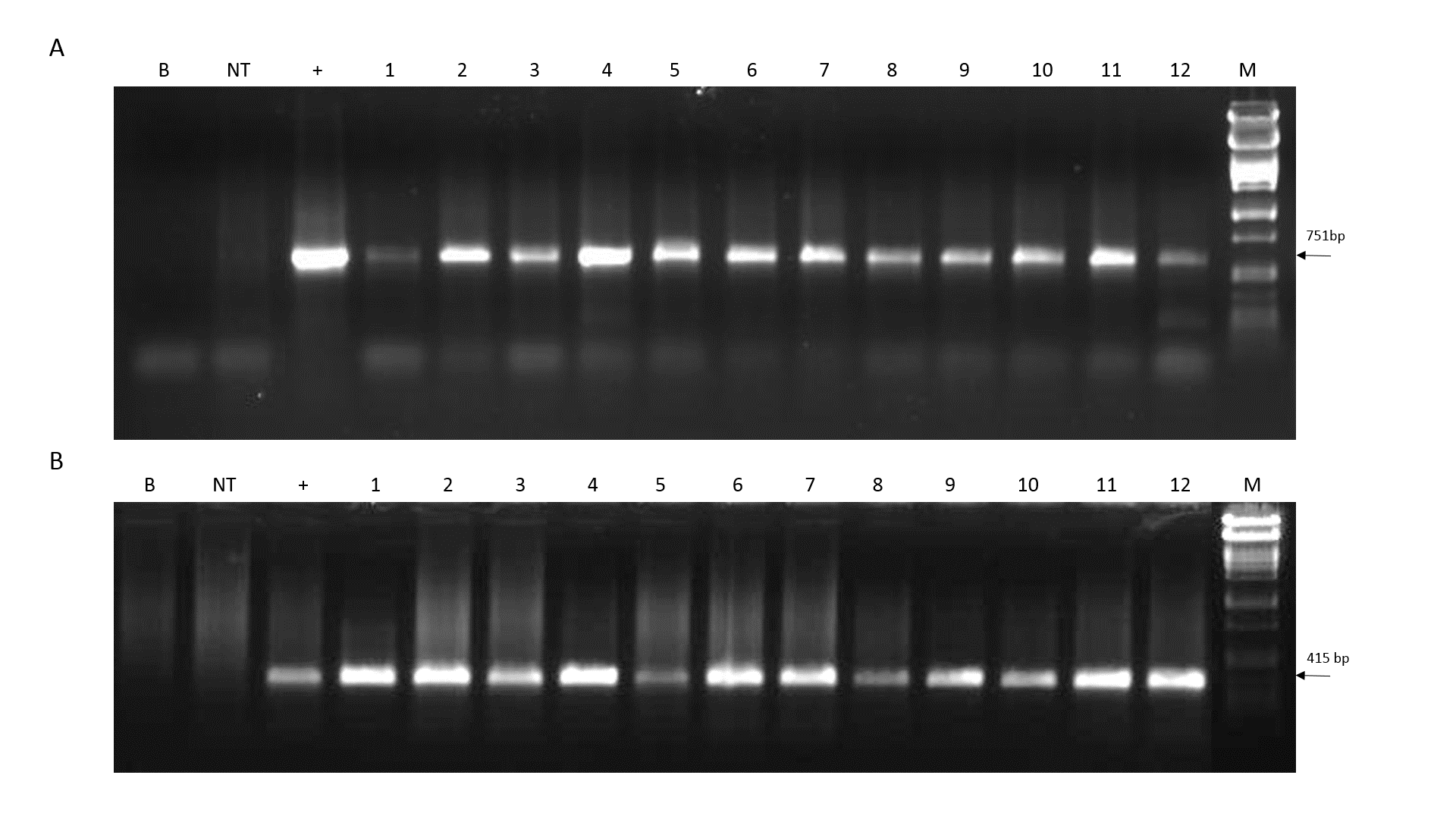


**Figure S3**. PCR analysis of the chimera events. (A) Cre-F/Cre-R primers and (B) HS-F/HS-R primers. B: blank; NT: non-transgenic Desiree, +: pCIP35, M: λ DNA (Gibco-BRL) digested with *Pst*I, and rests of lanes (1-12) are transgenic lines showing the required amplification (751 bp) and (415 bp) respectively.


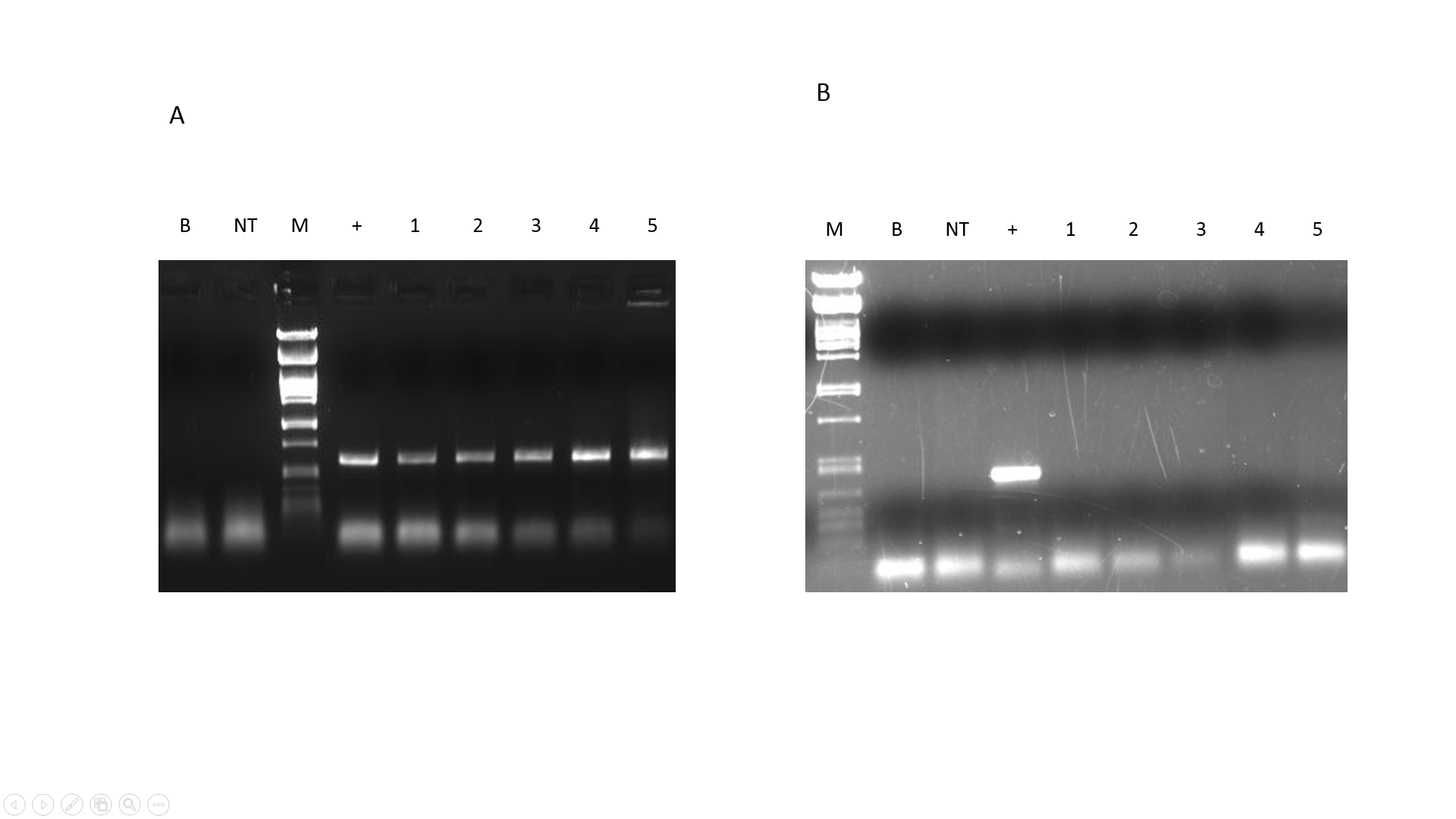


**Figure S4**. PCR analysis of the events without excision. (A) Cre-F/Cre-R primers and (B) HS-F/HS-R primers. B blank; NT non-transgenic Desiree; + pCIP35, M: λ DNA (Gibco-BRL) digested with *Pst*I, and rests of lanes (1-5) are transgenic lines showing the required amplification (751 bp) using Cre-F/Cre-R primers, but not using HS-F/HS-R primers.
