# Supplementary material for: Marker-free PLRV resistant potato mediated by Cre-loxP excision and RNAi
Source: Transgenic Res. 2016 Aug 20;25(6):813–28. doi: 10.1007/s11248-016-9976-y (PMC5104775; doi:10.1007/s11248-016-9976-y)
Supplement: Supplementary file 2 — Supplementary material 2 (PPTX 72 kb) [file 11248_2016_9976_MOESM2_ESM.pptx]

## Slide 1
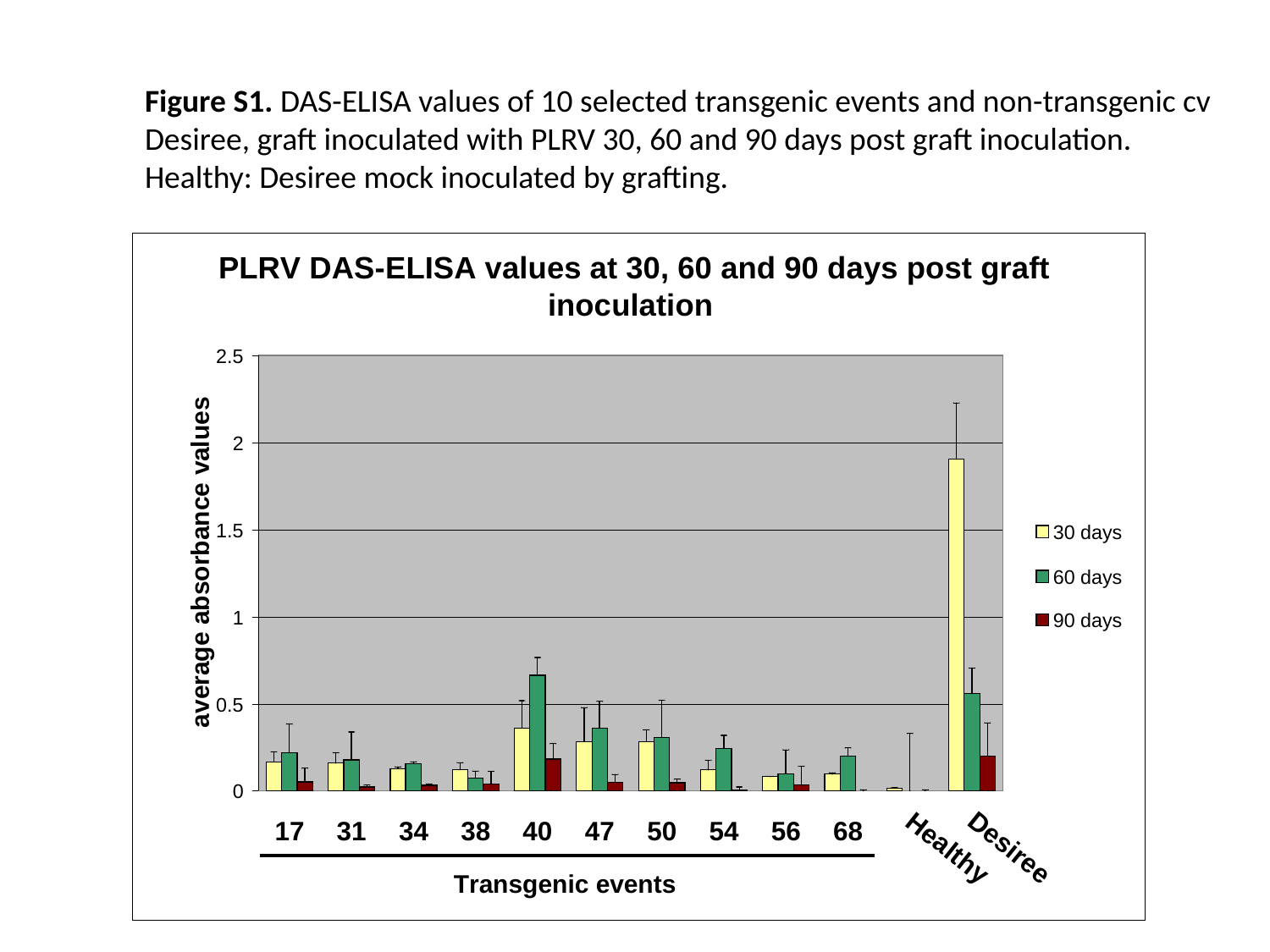

Figure S1. DAS-ELISA values of 10 selected transgenic events and non-transgenic cv Desiree, graft inoculated with PLRV 30, 60 and 90 days post graft inoculation. Healthy: Desiree mock inoculated by grafting.
